# Insight and challenges: mental health services in Nepal

**DOI:** 10.1192/bji.2020.58

**Published:** 2021-05

**Authors:** Yugesh Rai, Deoman Gurung, Kamal Gautam

**Affiliations:** 1 MD, Psychiatry Trainee, Essex Partnership University NHS Foundation Trust, UK. Email: raiyogesh39@gmail.com; 2 MRCPsych, ST4 (General Adult/Old Age Psychiatry), Lancashire Care NHS Foundation Trust, UK; 3 MD, Executive Manager and Consultant Psychiatrist, Transcultural Psychosocial Organization Nepal (TPO Nepal), Kathmandu, Nepal

**Keywords:** Nepal, mental health services, low- and middle-income countries, psychiatry, mental health

## Abstract

This paper describes the current state of mental health services in Nepal and reflects on the significant changes over the past decade. The main challenges to overcome are proper implementation of community-based services, the high suicide rate, stigma of mental illness, financial constraints, lack of mental health legislation and proper utilisation of human resources.

Nepal is a landlocked country situated in South Asia between India and China. It became a republic, federal state with the promulgation of the constitution in 2015. It is ethnically diverse, with 125 ethnic groups and approximately 123 languages spoken as mother tongues. The country has recently emerged from political transition and a massive earthquake. The current healthcare delivery system is organised as a tiered referral system. At the basic level are community health units, health posts, urban health clinics and primary hospitals (including primary health centres). More complex and serious cases are referred to secondary-level hospitals, tertiary-level hospitals (provincial and above) and eight specialised hospitals. The Ministry of Health and Population (MoHP) formulates overall health policies/plans and regulates, monitors and evaluates health activities and outcomes. In 2018, the Epidemiology and Disease Control Division (EDCD) of the Department of Health Services (DoHS) was designated as the focal unit to oversee mental health in Nepal. The mental health programmes in the country are operationalised by the Non-Communicable Disease and Mental Health Section.

## Mental healthcare system

Mental health services in Nepal started out in general hospital settings. The first psychiatric out-patient service started in 1962 and in-patient treatment in 1964.^1^ A mental hospital was established in 1984 and it moved to its current location in Lagankhel, Lalitpur, in 1985. It is the only mental hospital in Nepal and has a capacity of 50 beds. Nepal never had a mental asylum.

Mental health services are provided by the psychiatry units of medical colleges, provincial government hospitals and a few private hospitals. The total number of in-patient psychiatric facilities is 25 and the number of beds is 500. Clinics have been initiated in different subspecialties, such as child, memory, headache and addiction. The Child and Adolescent Psychiatry Unit at Kanti Children's Hospital is the only full-time out-patient clinic for children in Nepal. There is no dedicated in-patient unit for children.

Non-governmental organisations (NGOs) have played a vital role in the delivery of mental health services.^2^ Community mental health services were initiated in the 1980s by the United Mission to Nepal (UMN).^3^ In the 1990s and early 2000s, NGOs such as the Centre for Victims of Torture, Nepal (CVICT), the Centre for Mental Health and Counselling – Nepal (CMC-Nepal) and the Transcultural Psychosocial Organization Nepal (TPO Nepal) provided mental health and psychosocial care to the victims of civil conflict and the Bhutanese refugee crisis. NGOs have also contributed to the scaling up of community mental health programmes, in collaboration with the MoHP.

One of the hindrances to the development and delivery of mental healthcare is the lack of a social welfare net. Most mental healthcare is paid for out of pocket in Nepal. However, depression, psychosis, alcohol use disorder and epilepsy were recently included in the DoHS's Basic Health Service Package 2075 (2018). Thus, the care and treatment of these disorders will be free of cost. Medications included are diazepam, amitriptyline, chlorpromazine, trihexyphenidyl, phenobarbitone, carbamazepine, sodium valproate, risperidone and thiamine.

## Epidemiology of mental disorders

The first epidemiological field survey conducted in the Kathmandu Valley in 1984 estimated the prevalence of mental illness to be around 14%. A recent pilot study of the National Mental Health Survey reported the prevalence of mental disorders to be 12.9%.^3^ Suicide (16%) was the leading cause of death among women of reproductive age, with 21% of suicide occurring below the age of 18 years.^4^ In comparison with other countries, suicide among women (20 per 100 000) is higher than among men in Nepal (3rd highest cause of death among women versus 17th highest among men).^5^ The National Mental Health Survey is being conducted by the National Health Research Council in collaboration with the MoHP and World Health Organization (WHO) and is expected to be completed by January 2021.

## Stigma and cultural perception of mental illness

The mind and the body are considered distinct entities in Nepalese culture, thus mental illness is viewed as being separate from physical illness.^6^ Mental illness is perceived as a ‘spiritual dysfunction’ or ‘weak mind’ and attributed to spirit possession, black magic, divine wrath and misdeeds committed in previous lives (*karmako phal*). There is a strong belief in traditional healing and the first point of contact for most people is the traditional, religious or faith healers (e.g. *dhamis*, *jhankris*, *baidangis* and *bijuwas*).

Several emotional and somatic idioms of distress have been identified specific to the Nepalese cultural context.^7^ Emotional idioms are expressed as *dukkha lagyo* for sadness, *darr lagyo* for fear and *jharko laagyo* for irritation. Somatic idioms described as ‘gastric’ for abdominal pain and *jhum jhum* for tingling and numbness have been manifest in depressed patients attending healthcare facilities.

The family provides emotional and financial support and is actively involved in care. Key management decisions are usually made by senior family members. Stigma and discrimination towards people with mental illness is a major problem and mental health literacy is extremely low, resulting in hiding mental health problems, avoiding treatment and seeking alternative care. Stigma among service providers against people with mental illness has been identified as one of the barriers to mental healthcare. Involving patients and caregivers in treatment processes and building the capacity of non-specialist service providers can contribute to reducing stigma.^8^

## Training

Three-year postgraduate training in psychiatry (MD Psychiatry) started in 1997 and is now available in 16 institutions. There are five different post-graduate training programmes, but the training curriculum and evaluation process is not uniform. There are currently about 45 residents in psychiatry training. The undergraduate syllabus in psychiatry is not nationally standardised and each university has its own version.^9^ The number of psychiatrists has grown from 40 in 2008 to 200 at present. Similarly, there has also been growth in the numbers of clinical psychologists and other mental health professionals. Details of the mental health workforce are presented in Table 1.

**Table 1:** Mental health services and resources in Nepal

	2020	2008^10^
Health budget as a proportion of national budget	6.15%	6.5%
Mental health budget as a proportion of the total health budget	0.2%	0.8%
Number of registered doctors in Nepal	26 346	6719
Number of psychiatrists	200	39
Number of in-patient psychiatric facilities	25	–
Number of psychiatric beds	500	385
Child psychiatrists	3	0
Old age psychiatrists	0	0
Clinical psychologists	30 (MPhil) 200 (MA)	>9
Psychiatric nurses	50	48
Psychosocial counsellors (trained using the 780 h curriculum of NGOs)	700	–
Community-based psychosocial workers (trained in basic emotional support)	>300	–

NGO, non-governmental organisation; –, not reported.

## Mental health policy and legislation

A comprehensive National Mental Health Policy was first formulated in 1996 and incorporated in the Ninth Five Year National Plan by the Government of Nepal.^9^ However, the implementation of the policy was ineffective, and the Mental Health Act never came into existence. Several attempts were made to revise the policy and ensure effective implementation. The EDCD prepared a draft in 2018, which has undergone rigorous consultations with federal, provincial and local government representatives in mental health and is planned to be endorsed through the MoHP. The five key strategies are:

(a) to ensure the availability and accessibility of optimal mental health services for all the population of Nepal
(b) to ensure management of essential human and other resources to deliver mental health and psychosocial services
(c) to raise awareness of mental health to demystify mental illness and reduce associated stigma and promote mental health
(d) to protect the fundamental rights of people with psychosocial disability and mental illness
(e) to promote and manage health information systems and research in mental health programmes.

The MoHP developed the Community Mental Health Care Package Nepal, 2074 in 2017 to facilitate implementation of the 1996 National Mental Health Policy.^11^ This package is guided by the principles of integration of mental health into primary care and the WHO Mental Health Gap Action Programme (mhGAP). The MoHP has been scaling up nationwide community mental health programmes based on this package. Similarly, the National Health Training Centre of the MoHP has developed four different training modules for building the capacity of non-specialist service providers.^12^

## Psychiatric association

The Psychiatrists’ Association of Nepal (PAN) is non-profit professional organisation of Nepalese psychiatrists established in 1990.^9^ It regularly organises annual meetings and educational events. It publishes a biannual peer-reviewed journal (*Journal of Psychiatrists’ Association of Nepal*). Recently, PAN has been active in the national mental health programmes, policy reforms and advocacy.

## The way forward

Psychiatric services have seen tremendous developments over the past decade, such as the increased number of mental health professionals, scaling up of community mental health programmes, establishment of the country's first child and adolescent psychiatry unit, designation of a focal mental health unit at the MoHP, initiation of the National Mental Health Survey and active engagement of PAN. Despite these improvements, the following challenges need to be addressed urgently.

(a) The budget allotted for mental health is still low, and there is a need to increase the budget to ensure effective scaling up of community-based mental health programmes throughout the country.
(b) Lack of awareness on mental health and prevailing stigma have been key barriers to accessing mental healthcare. This demands the formulation and implementation of awareness-raising and anti-stigma campaigns in communities.
(c) There is a need to fill existing vacancies; increase recruitment of psychiatrists; and create positions for clinical psychologists, psychiatric nurses, psychosocial counsellors and community-based psychosocial workers in the government healthcare system.
(d) The need for subspecialties in psychiatry is emerging over time. The government, universities and medical colleges should envision initiating various subspecialty programmes in child and adolescent, geriatric, addiction and forensic psychiatry.
(e) The suicide rate has been a grave concern but there is no national suicide registry or suicide-prevention strategy in Nepal. A mechanism for suicide reporting and surveillance and interventions to reduce suicide need to be developed and implemented at a national level.
(f) Scientific research and the generation of evidence on mental health and illness in Nepal is predominantly reliant on NGOs. The government should prioritise mental health research via academic universities and teaching hospitals.
(g) Although community mental health programmes have been scaled up, there is lack of clinical supervision of trained non-specialist service providers and no regular supply of psychotropic medications.^12^ These need to be ensured for effective implementation of mental health services in primary care settings.
